# Placenta Percreta Progression to Resistance Against Uterine Artery Embolization and Penetration Into the Bladder

**DOI:** 10.7759/cureus.55651

**Published:** 2024-03-06

**Authors:** Yukiko Miyashita, Tasuku Mariya, Masayuki Someya, Shinichi Ishioka, Tsuyoshi Saito

**Affiliations:** 1 Department of Obstetrics and Gynecology, Sapporo Medical University, Sapporo, JPN

**Keywords:** uterine artery embolization (uae), interventional radiology guided embolization, vesicouterine fistula, placenta previa, placenta percreta

## Abstract

A 31-year-old female sought termination of pregnancy due to a fetal body stalk anomaly diagnosed at 18 weeks of gestation. Despite an anterior placenta previa, successful vaginal delivery occurred. However, placental adhesion over a previous cesarean scar occurred, and part of the placenta could not be removed. Immediate postpartum bleeding prompted imaging studies, revealing extravasation from adherent placental remnants. Uterine artery embolization (UAE) provided initial hemostasis, but recurrent bleeding necessitated re-embolization. Although conservative treatment was initially pursued, significant hematuria prompted reevaluation, revealing extensive uterine wall and bladder penetration. Surgical intervention with total hysterectomy and partial bladder resection was performed, leading to the successful recovery of bladder function following surgical repair. While this case achieved a positive outcome, there is a potential for permanent urinary dysfunction if lesions are more extensive. While achieving a conservative cure is ideal, it is essential to assess the timing for opting for surgical intervention.

## Introduction

The definitive treatment for placenta percreta involves the complete removal of the placenta, usually accompanied by a total hysterectomy [1]. Therefore, providing treatment for patients with placenta percreta who desire fertility preservation is often challenging. As a conservative treatment, uterine artery embolization (UAE) to control genital bleeding from a placental remnant is often attempted [2,3]. In the present case, a patient expressed a strong desire for uterine preservation despite experiencing massive genital bleeding from remnants of placenta percreta. However, multiple pelvic embolizations resulted in complete bladder perforation and needed surgical intervention. This case report aims to provide a detailed clinical course of the case's progression and reconsider the therapeutic intervention strategy for this specific case.

## Case presentation

**Figure 1:**
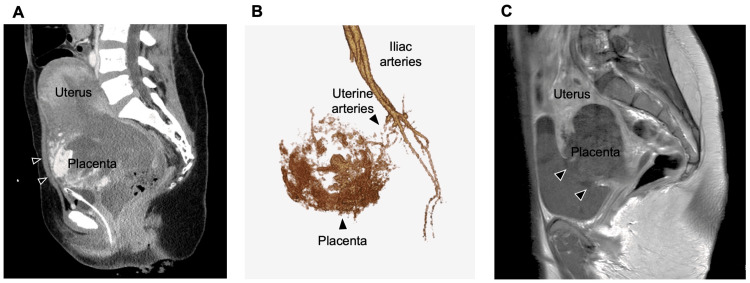
Imaging evaluation of the residual placenta and penetration into the bladder A. Enhanced CT image shows active extravasation from the residual placenta (arrow). B. 3D-CT angiography image of the hypervasculated placenta fed by the uterine artery. C. MRI of perforation of the placenta into the bladder (arrow) and clot retention in the bladder

The case is a 31-year-old female (gravida 4, para 2) who had undergone two cesarean section deliveries. She desired termination of pregnancy due to a fetal body stalk anomaly diagnosed by ultrasound at 18 weeks of gestation [4]. Although she had an anterior placenta previa of grade IV, complete previa, successful vaginal delivery of the fetus was achieved. However, the placenta covered the cesarean scar of the previous pregnancy, resulting in a placental remnant. Despite the absence of antenatal ultrasound findings indicative of the placenta accreta spectrum, a part of the placenta could not be removed. On the day after delivery, massive genital bleeding was observed, prompting further imaging examination. Contrast-enhanced CT revealed extravasation from the adherent placental remnants (Figure 1A), and 3D-CT angiography demonstrated abundant blood flow to the placenta through the uterine arteries (Figure 1B). Hemostasis was promptly achieved through UAE with an absorbable gelatin sponge, but three weeks later, there was a recurrence of massive genital bleeding and hematuria. Re-embolization extending to the branches of the internal iliac artery was performed, and successful hemostasis was achieved. Many reports have shown successful conservative treatment of the placenta accreta spectrum, even in percreta, with UAE [2,3]. Although we initially hoped for a conservative resolution in the case, the patient showed significant hematuria two weeks after the second embolization. Evaluation with contrast-enhanced MRI revealed an extensive defect of the uterine wall in the adherent placental region, with complete penetration into the bladder (Figure 1C).

**Figure 2:**
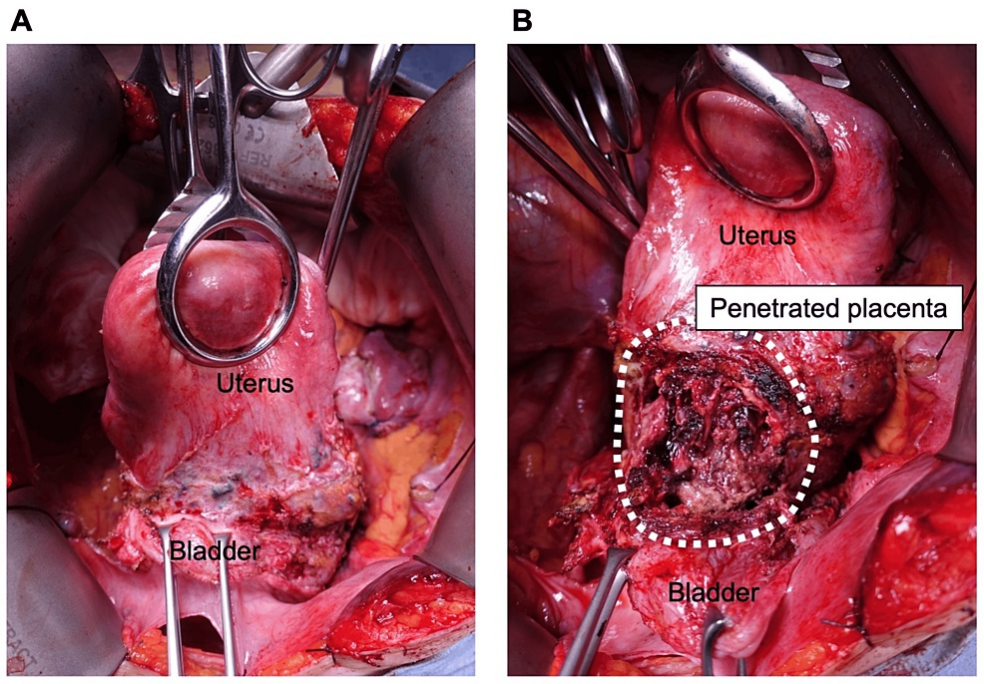
Surgical findings at the time of hysterectomy A. Process of bladder dissection from the uterus. B. Placental penetration exposed after complete dissection of the bladder from the uterus

Conservative treatment was deemed unfeasible, and a surgical approach was taken with total hysterectomy and partial bladder resection and repairment. The cesarean scar was completely covered by the bladder (Figure 2A), and upon dissection, a significant portion of the bladder muscle layer was found to be absent (Figure 2B). While the perforation site could be surgically closed, it took several months for bladder function to recover.

## Discussion

Cesarean section should be chosen for deliveries with placenta previa. However, cesarean section is not always the best choice in all cases of termination of pregnancy. Several articles have shown the safety of vaginal delivery in the termination of pregnancy with placenta previa [5,6]. Most placenta previa cases are successfully delivered vaginally without hysterectomy or blood transfusion, although some cases require UAE for hemostasis or prophylactic use [7]. Prophylactic UAE also could be considered in the present case before termination of pregnancy, but progressive bladder penetration would probably have been unavoidable even in that case. In this case, it is believed that there was a progressive viable placental invasion resistant to UAE. However, it is also important to consider the ischemic effects associated with multiple embolizations. The bladder is known to be an organ that is sensitive to ischemia, and there have been several reports of bladder necrosis caused by UAE and other pelvic artery embolization [8,9]. However, in a report on bladder necrosis following UAE for placental accreta, it was necessary to excise the necrotic tissue during the repair process. Additionally, histological confirmation of necrotic bladder tissue was obtained [10]. In the present case, although histologically there was adherence of bladder tissue to the excised uterus and placenta, bladder necrosis was not evident. Additionally, despite repeated UAE for placenta accreta in the first trimester, a case report states that no particular problems were noted. They also emphasize the importance of multiple embolizations in severe cases to achieve sufficient vessel occlusion, particularly for placental tissue, which is often resistant to hypoxia [11]. Therefore, we conclude that the bladder penetration following pelvic embolization observed in our case is primarily attributed to the invasion of residual viable placental tissue.

## Conclusions

Fortunately, bladder function was eventually restored after surgical repair in this case. However, if the extent of the damaged lesion had been more extensive, there might have been permanent urinary dysfunction. The timing of the decision to proceed with surgical treatment is crucial.
